# SIRT6 promotes angiogenesis and hemorrhage of carotid plaque via regulating HIF-1α and reactive oxygen species

**DOI:** 10.1038/s41419-020-03372-2

**Published:** 2021-01-12

**Authors:** Zhou Yang, Yijun Huang, Lei Zhu, Kai Yang, Kun Liang, Jinyun Tan, Bo Yu

**Affiliations:** 1 Department of General Surgery, Shanghai Pudong Hospital, Fudan University Pudong Medical Center, Shanghai, 201399 China; 2 Department of General Surgery, Huashan Hospital North, Fudan University, Shanghai, 201907 China; 3 Department of Vascular Surgery, Huashan Hospital, Fudan University, Shanghai, 200040 China

**Keywords:** Gene therapy, Cell biology

## Abstract

As a member of Sirtuins family, SIRT6 participates in the physiological and pathological progress of DNA repair, anti-aging, metabolism, and so on. Several studies have demonstrated that knockdown of SIRT6 inhibited the development of atherosclerosis (AS), indicated SIRT6 as a protective factor for AS. However, we confirmed SIRT6 was significantly overexpressed in human unstable carotid plaques compared with stable carotid plaques. This result indicated a more complex role of SIRT6 in AS. Furthermore, we constructed mice model with unstable carotid plaque and injected them with SIRT6 overexpressed adeno-associated virus (AAV-SIRT6). AAV-SIRT6 significantly promoted angiogenesis as well as hemorrhage in plaques. In vitro, we demonstrated overexpression of SIRT6 prevented HIF-1α from degradation by deubiquitination at K37 and K532 of HIF-1α, thus promoted the expression of HIF-1α under both normoxia and hypoxia in human umbilical vein endothelial cells (HUVECs). Through regulating HIF-1α, overexpression of SIRT6 promoted invasion, migration, proliferation, as well as tube formation ability of HUVECs. Interestingly, under different conditions, SIRT6 played different roles in the function of HUVECs. Under oxidative stress, another important pathological environment for AS, SIRT6 bound to the promoter of Catalase, a main reactive oxygen species scavenger, and depleted H3K56 acetylation, thus inhibited expression and activity of Catalase at the transcriptional level. Subsequently, inhibited Catalase promoted reactive oxygen species (ROS) under oxidative stress. Accumulated ROS further aggravated oxidative stress injury of HUVECs. On one hand, SIRT6 promoted angiogenesis in plaque via HIF-1α under hypoxia. On the other hand, SIRT6 promoted injury of neovascular via ROS under oxidative stress. It is this process of continuous growth and damage that leads to hemorrhage in carotid plaque. In conclusion, we innovatively confirmed SIRT6 promoted the angiogenesis and IPH via promoting HIF-1α and ROS in different environments, thus disclosed the unknowing danger of SIRT6.

## Introduction

Cardiovascular disease is the leading killer of human death, in which AS is the ringleader. Furthermore, carotid AS accounts for about 25% of all atherosclerotic lesions, frequently occurs at the bifurcation of the carotid artery and is closely related to stroke. Vulnerable rupture, secondary thrombosis or embolism are important causes of stroke^1^. Plaque neovascularization and accompanied intraplaque hemorrhage (IPH) is an important histological feature of unstable plaques, whereas the molecular mechanism of them remains unclear^2,3^.

The Sirtuins family is Class III deacetylases relying on nicotinamide adenine (NAD+). They play important roles in metabolism, aging, and so on. Among them, SIRT6 is a nuclear histone deacetylase that specifically targets H3K56ac and H3K9ac^4,5^. Most previous studies suggested SIRT6 works as a protective factor of AS. In apolipoprotein E-deficient mice (ApoE(−/−) mice), the deletion of SIRT6 via lentivirus injection accelerated endothelial dysfunction and atherosclerosis^6^. Similarly, Sirt6(+/−) ApoE(−/−) mice show increased plaque formation, which may result from increased expression of NKG2D ligands and subsequently increased cytokine expression^7^. However, the research in the overexpression of SIRT6 was deficient. Only found one study confirmed that overexpression of SIRT6 in human endothelial cells (ECs) inhibits monocyte adhesion to endothelial cells e in vitro^8^. It may not enough to explain the complex role of SIRT6 in AS.

In this study, we comprehensively investigated the role of SIRT6 from clinical specimens, HUVECs under different conditions, as well as unstable carotid plaque mice model to learn the effect of SIRT6 at carotid AS. We innovatively confirmed SIRT6 was associated with the stability of carotid plaque and disclosed the unknowing danger of SIRT6.

## Materials and methods

### Patients and specimens

The specimens of carotid plaque were acquired from 36 patients undergoing carotid endarterectomy (CEA) at Huashan Hospital between August 2018 and July 2019 (Table 1). Patients with the following criteria recruited: carotid stenosis of 50–99% with stroke; transient ischemic attack (TIA) symptoms in the past 6 months or carotid stenosis of 60–99% without stroke; TIA symptoms in the past 6 months. The Institutional Ethical Review Boards of Huashan Hospital at Fudan University approved this research. All participants gave their written informed consent before surgery. In addition, patients were divided as stable group and vulnerable group based on the following standards as described in previous research^9^.

**Table 1:** Characteristics carotid AS patients.

Characteristics	Number of cases
*Stability*	
Stable	14
Unstable	22
*Age (y)*	
≥65	15
<65	21
*Gender*	
Male	26
Female	10
*BMI (kg/m*^*2*^*)*	
≤25	24
>25	12
*Symptom*	
Yes	19
No	17
*Stenosis*	
≤90%	13
>90%	13
*Hypertension*	
Yes	31
No	5
*Hyperlipidemia*	
Yes	7
No	29
*Ischemic heart disease*	
Yes	11
No	25
*Diabetes*	
Yes	12
No	24

### Evaluation of vascular density in plaques

The evaluation of vascular density in plaque was analyzed as microvascular density (MVD) previously described^10^. The vessels in plaque were labeled by IHC of CD31. The area of densest plaque neovascularization (hot plot) was identified in each plaque under low-power lens (magnification: ×100). Subsequently, at least 5 high-power fields in the hot plot were captured (magnification: ×400). Any single cell or cell mass stained with CD31, as long as it has a clear separation from the surrounding cells, it is considered to be a countable microvascular. The average microvascular number of at least 5 fields was identified as the MVD of the plaque.

### Regents, cell culture, hypoxia, and oxidative stress induce

ROS scavenger N-acetyl-L-cysteine (NAC), proteasome inhibitor MG-132, and HIF-1α inhibitor YC-1 were all purchased from MedChemExpress, Inc. (Monmouth Junction, NJ, USA). Cobalt chloride (CoCl_2_) was purchased from Sigma-Aldrich, Inc. (Burlington, MA, USA). The work concentration and time were confirmed by instructions combined with experimental requirements.

HUVECs were purchased from Allcells, Inc. (Alameda, CA, USA) and cultured in Endothelial Cell Medium (ECM; ScienCell Research Laboratories, Carlsbad, CA, USA) supplemented with 10% FBS (Invitrogen, Carlsbad, CA, USA). Human vascular smooth muscle cells (T/G HA-VSMC) and human renal epithelial cell line HEK-293T were purchased from the University of Colorado Cancer Center Cell Bank and cultured in RPMI 1640 medium supplemented with 10% FBS. All cells were cultured at 37 °C in a 5% CO_2_ atmosphere.

For hypoxia induce, cells were treated with cobalt chloride (CoCl_2_, 200 μM for 24 h) which prevents HIF-1α from degeneration by replacing the prolyl hydroxylase (PHD) cofactor Fe^2+^ ^11^.

### LC-MS/MS analysis

To identify the ubiquitination sites of HIF-1α, HUVECs were co-transfected with Flag-SIRT6, Myc-HIF-1α, and HA-Ub, respectively, followed by MG-132 (10 μM) for 6 h before being harvested for assays. Myc-HIF-1α was pulled down by Co-IP with an anti-Myc antibody. The IP production was separated by SDS-PAGE and digested by trypsin. The resulting tryptic peptides were extracted and subjected to electrospray ionization quadrupole time-of-flight (Orbitrap Fusion Lumos) mass spectrometry (MS). MS full scans were acquired in Data Analysis (Thermo Finnigan, San Jose, CA). Peptides were identified from MS/MS spectra by searching against SwissProt database (2015_04) using the Mascot search algorithm23.

### Mutant plasmids construction and transfection

pcDNA3.1-SIRT6-Flag, pcDNA3.1-Ub-HA, and pcDNA3.1-HIF1A-Myc plasmids were purchased from HedgehogBio, Inc. (Shanghai, China). The mutant vectors (pcDNA3.1-SIRT6-H133Y-Flag, pcDNA3.1-Ub-K48R/K63R-HA, pcDNA3.1-HIF1A-K37R/K477R/K532R/K538R/K547R-Myc) were constructed by QuickMutation™ assay kit (Beyotime Biotechnology, Shanghai, China).

For transfection, 10^6^ cells were seeded in 6-cm dish and cultured at 37 °C. After 18 h, 2 μg plasmid accompanied with 10 μl Lipofectamine 3000 (Invitrogen, Inc.) were added in culture media. Subsequently, cells were further cultured at 37 °C for 36 h.

### Determination of ubiquitination by co-immunoprecipitation (Co-IP)

10^7^ cells (transfected with different pairs of plasmids described above) were harvested and lysed using NP-40 buffer. Lysates were pre-cleared by 20 μL Protein A/G sepharose beads (1:1000, Santa Cruz, CA, USA) and centrifuged for supernatant. The pre-cleared lysate was added with 1 μg negative control IgG (Abclonal), anti-HIF-1α or anti-Myc-tag rabbit polyclonal antibody (ProteinTech Group. Inc., Wuhan, China), incubated for 12 h at 4 °C while rotating. Further 50 μl Protein A/G sepharose bead was added in lysate to capture the immunocomplex. After incubating for 4 h at 4 °C, the beads were harvested by centrifugation at 3000 × *g* for 3 min, and washed four times with NP-40 buffer. Elution of the proteins was conducted by adding 2× SDS loading buffer to the beads and boiling for 5 min at 95 °C. Subsequent, western blot was performed as described below with anti-Ub or anti-HA-tag rabbit polyclonal antibodies.

### Detection of Catalase, superoxide dismutase, and glutathione peroxidase enzyme activity

All three enzymes activity was measured by assay kits provided by Beyotime Biotechnology. Catalase can catalyze hydrogen peroxide to produce water and oxygen. The residual hydrogen peroxide can oxidize the chromogenic substrate under the catalysis of peroxidase to produce the N-(4-antipyryl)-3-chloro-5-sulfonate-p-benzoquinone monoamine, with the maximum absorption wavelength of 520 nm (OD520). Based on this principle, cells were lysed with RIPA lysis buffer and reacted with chromogenic substrate. Meanwhile, standard curve was made by hydrogen peroxide standard. The OD520 was measured by Microplate Reader (Tecan, San Jose, CA, USA) and put into standard curve to acquire the final activity of Catalase.

Similarly, superoxide dismutase and glutathione peroxidase enzyme activity were also measured as described above via reaction with corresponding substrate. All data were normalized by protein concentration via BCA assay as described above.

### Chromatin immunoprecipitation (CHIP) assay

CHIP assay was performed according to the manufacturers’ instructions by using Magna ChIP Kit (Millipore, Bedford, MA, USA). Chromatin samples were immunoprecipitated with antibodies against a negative control normal rabbit IgG, H3K56ac (Active motif, Carlsbad, CA, USA) or SIRT6 (Abcam), respectively. Subsequently, IP production was performed with RT-qPCR as described above. The primers of NRROS promoter were as follows: Forward: 5′‑TAGCTATGGAGCGCAAGGC‑3′ and reverse: 5′‑TATCAGCATCCTTCAGGCCG‑3′.

### Matrigel plug assay

Animal studies were performed according to the guidelines from Directive 2010/63/EU of the European Parliament on the protection of animals used for scientific purposes. All detailed experimental procedures were approved by the Institutional Animal Care and Utilization Committee of Fudan University Pudong Animal Experimental Center.

In Matrigel plug assay, we set up four groups: Blank, SIRT6 activator (UBCS039, 100 µM), SIRT6 activator+HIF-1α inhibitor (YC-1, 2 µM), SIRT6 activator+ ROS scavengers (NAC, 10 mM). All drugs were suspended in the Matrigel before injection. Eight-week-old C57Bl6 mice (provided by Model Animal Research Center of Nanjing University) were anesthetized with isoflurane (4% for induction, 2% isoflurane for maintenance) and 0.5 mL ice-cold Matrigel (growth factors reduced, BD Biosciences) supplemented with bFGF (50 ng/ml, R&D Systems, Inc., Minneapolis, MN, USA) was injected into the abdominal subcutaneous tissue (3 mice per group, each mice was injected 2 Matrigel plugs). After 14 days, the animals were injected with 100 µl FITC-dextran (2 × 10^6^ MW) accompanied with TRITC-dextran (4400 MW, 10 mg/ml dissolved in PBS, 1:1 ratio) via tail vein. Large (2 × 10^6^ MW) fluorescein isothiocyanate (FITC)-labeled dextran identifies functional vessels within Matrigel, while smaller (4400 MW) tetramethylrhodamine (TRITC)-labeled dextran can be used to highlight the permeability of these vessels^12^. After 15 min, mice were euthanized by rapid cervical vertebra dislocation. The Matrigel plugs were applicated in IHC and leakage ratio analysis. For IHC analysis, expression of HIF-1α and CD31 was measured. IHC of CD31 was further applicated to assess MVD in Matrigel plugs. In addition, the ratio of TRITC-dextran/FITC-dextran fluorescence area was analyzed as leakage ratio of vessels.

### Establishment of a mice model with carotid plaque

Seven-week-old male ApoE (−/−) mice were provided by the Model Animal Research Center of Nanjing University. All the mice (*n* = 24) were equally and randomly divided into the Blank, Negative Control Model, SIRT6 Overexpressed Model group. The Blank group was fed with a high-fat diet (HFD; Research Diets, Inc., New Brunswick, NJ, USA) for 13 weeks without surgical model construction. The Negative Control Model and SIRT6 Overexpressed Model were both fed with HFD for 5 weeks, and injected with 10^11^vg adeno-associated virus serotype 9: AAV-NC and AAV-SIRT6 diluted in 100 μl PBS, respectively, via tail vein (Generated by HanBio, Inc., Shanghai, China). Supplemented with one more week HFD feeding, these two groups were performed with operation for the construction of unstable plaque model as described below. Subsequently, these two mice models were fed with HDF until the 13th week. At 13th week, all mice in three groups were anaesthetized with 1% sodium pentobarbital and drawn blood from heart. Subsequently, the carotid artery accompanied by plaque mice were harvested after 4% paraformaldehyde perfusion.

The mice model with carotid plaque was generated by tandem stenosis reported previously^13^. Briefly, mice were anesthetized with isoflurane (4% for induction, 2% isoflurane for maintenance), the right common carotid artery and bifurcation were separated. The first knot was tied under the carotid bifurcation by 1 mm with 6-0 polyester thread. Subsequently, the second knot was tied at 3 mm below the first knot. Finally, the incision is cleaned with normal saline, the skin is sutured.

### Statistical analysis

All the experiments were performed three times at least. SPSS software (version 19.0, IBM Corp., Armonk, NY, USA) was used for statistical analysis of all the experimental data. GraphPad Prism (version 7, GraphPad Software, La Jolla, CA, USA) was used to determine the statistical results. All data are expressed as the mean + standard deviation (mean + sd). The statistical analysis of the data from two groups was performed using a *t*-test. The comparisons of multiple groups were performed by one-way ANOVA and then an LSD *t*-test. *P* < 0.05 was considered to be significant.

## Results

### SIRT6 was overexpressed in unstable carotid plaque accompanied by co-location of HIF-1α

**Fig. 1:**
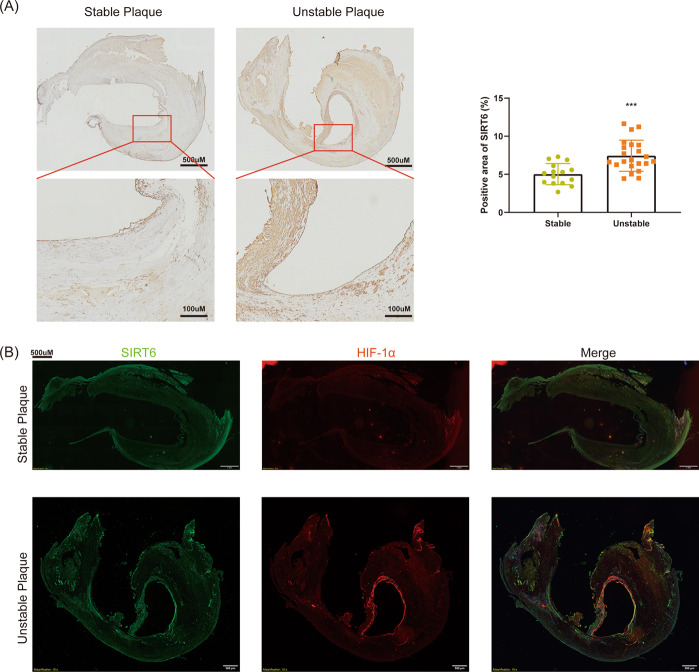
SIRT6 was overexpressed in unstable carotid plaque accompanied by co-location of HIF-1α. **A** IHC of SIRT6 in stable (*n* = 14) and unstable carotid plaques (*n* = 22). **B** Co-location of SIRT6 and HIF-1α in stable and unstable carotid plaques (***P < 0.001).

Previous research rarely investigated the expression of SIRT6 in clinical plaque specimens. We first measured the expression of SIRT6 in stable and unstable carotid plaques derived from CEA, respectively. We found SIRT6 was significantly upregulated in unstable carotid plaques compared with stable carotid plaques, which indicated SIRT6 may be associated with the stability of carotid plaques (Fig. 1A). In our previous research, we demonstrated SIRT6 interacted with HIF-1α in thyroid cancer cells. HIF-1α is an important transcriptional factor associated with angiogenesis, which is also confirmed to be risk factors for AS^14^. Interestingly, we found SIRT6 was also co-located with HIF-1α in both unstable carotid plaques and stable carotid plaques (Fig. 1B). Therefore, we suggested SIRT6 may regulated the stability of carotid plaques via interacting with HIF-1α.

### Overexpression of SIRT6 promoted angiogenesis and hemorrhage in carotid plaque

**Fig. 2:**
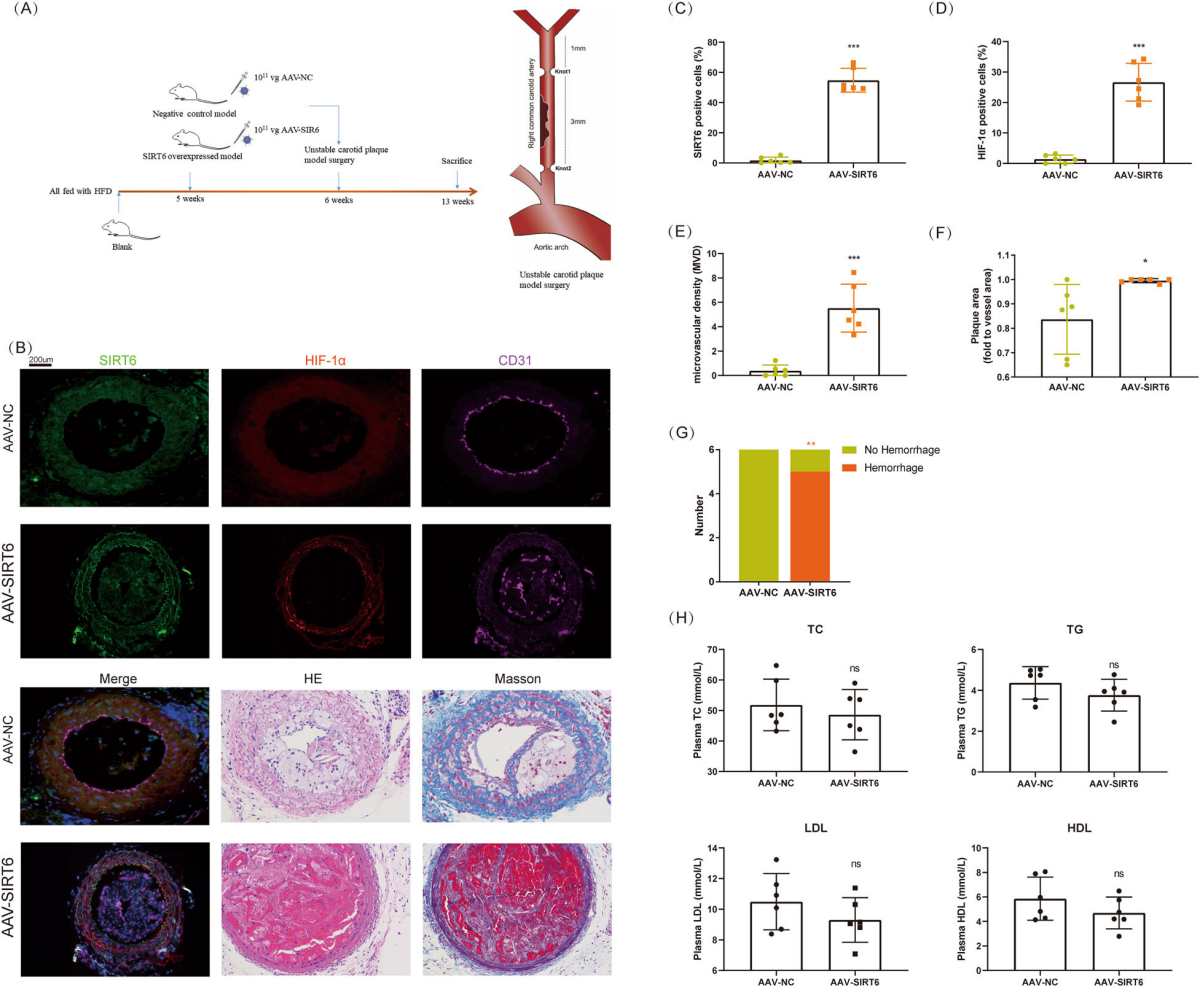
Overexpression of SIRT6 promoted angiogenesis and hemorrhage in carotid plaque. **A** Establishment of a mice model with unstable carotid plaque. SIRT6 was overexpressed by adenovirus associated virus (*n* = 6). **B** Immunofluorescence of SIRT6, HIF-1α, and CD31; HE staining; Masson staining of carotid plaque in mice model. **C** Analysis of the expression of SIRT6 in carotid plaque. **D** Analysis of the expression of HIF-1α in carotid plaque. **E** Microvascular density (MVD, marked by CD31) in carotid plaque. **F**, **G** Plaque area and hemorrhage analyzed by HE staining and Masson staining. **H** Quantification of total cholesterol (TC), triglyceride (TG), low-density lipoprotein (LDL), and high-density lipoprotein (HDL) in serum of mouse model (ns: no significance, *P < 0.05, **P < 0.01, ***P < 0.001).

To determine the role of SIRT6 in vivo, we constructed a mice model with carotid plaque. AAV was adopted for the overexpression of SIRT6 (Fig. 2A, B, C). We found overexpression of SIRT6 significantly increased the expression of HIF-1α and angiogenesis marker CD31 (analyzed as MVD) in plaque (Fig. 2B, D, E). Furthermore, the overexpression of SIRT6 also promoted the plaque area and ratio of IPH (Fig. 2B, F, G). However, SIRT6 showed no effect in main plasma cholesterol indexes, which are also important risk factors for AS (Fig. 2H). The constructed model successfully confirmed SIRT6 promoted expression of HIF-1α, angiogenesis, and IPH of carotid plaque. As endothelial cell is the main target of angiogenesis, we further investigated the location of SIRT6 and CD31 (the marker for endothelial cell) in unmodeled ApoE(−/−) mice (endothelial cells were severely absent in model mice and clinical specimens). SIRT6 and CD31 were obviously co-located, thus we focused our research in HUVECs (Figure S1).

### SIRT6 increased the expression of HIF-1α at protein level under both normoxia and hypoxia

**Fig. 3:**
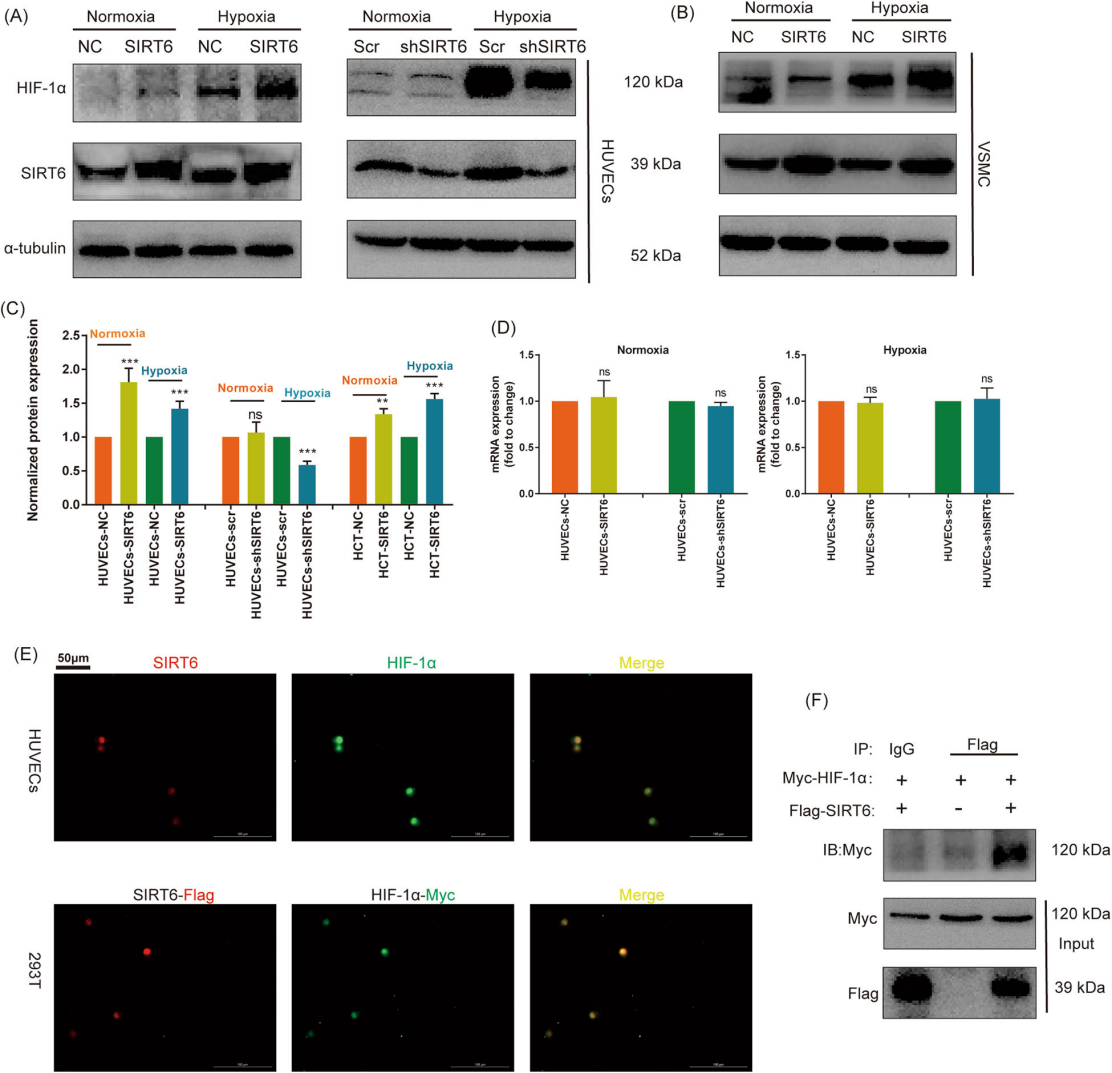
SIRT6 promoted the expression of HIF-1α in HUVECs under both normoxia and hypoxia. **A** The expression of HIF-1α under hypoxia (CoCl_2_) and normoxia after overexpression and downregulation of SIRT6 in HUVECs. **B** The expression of HIF-1α under hypoxia (CoCl_2_) and normoxia after overexpression of SIRT6 in VSMC. **C** Quantitative densitometric analysis of HIF-1α levels in panels **A** and **B**. **D** The mRNA expression of HIF-1α under hypoxia and normoxia after overexpression and downregulation of SIRT6 in HUVECs performed by RT-qPCR. **E** Colocalization of HIF-1α (Myc-tag) and SIRT6 (Flag-tag) under hypoxia in HUVECs and 293T performed by IF (magnification: ×200). **F** Co-IP of HIF-1α-Myc and SIRT6-Flag under hypoxia in 293T cell (ns: no significance, ***P < 0.001).

To investigate the effect of SIRT6 at HIF-1α, SIRT6 stably overexpressed/downregulated HUVECs and their negative control HUVECs were constructed. Interestingly, we found overexpression of SIRT6 significantly increased the protein expression of HIF-1α under both normoxia and hypoxia. Meanwhile, the downregulation of SIRT6 decreased the expression of HIF-1α only under hypoxia, with possible absence of decreased expression of HIF-1α in normoxia (Fig. 3A, C). We inferred HIF-1α in normoxia was already unstable, and downregulation of SIRT6 was difficult to further promote its degradation. In addition, overexpression of SIRT6 in VSMC also promoted the expression of HIF-1α under both normoxia and hypoxia (Fig. 3B, C). Subsequently, we further investigated the mRNA expression in HUVECs and found overexpression of SIRT6 showed no change in the mRNA expression of HIF-1α under both normoxia and hypoxia (Fig. 3D). Therefore, we suggested SIRT6 regulated HIF-1α at the protein level but not the transcription level. Both SIRT6 and HIF-1α are nucleoprotein, thus we suggested they are interacted. We investigated the location of SIRT6 and HIF-1α under hypoxia by IF and found an obvious colocalization. Meanwhile, co-transfection of SIRT6-Flag and HIF-1α-Myc plasmids in 293T cells detected by tagged antibodies (Flag/Myc) also showed similar results (Fig. 3E). Finally, Co-IP assay confirmed the significant co-precipitation of HIF-1α-Myc with SIRT6-Flag (Fig. 3F).

### Overexpression of SIRT6 inhibited ubiquitin-proteasome degradation of HIF-1α

**Fig. 4:**
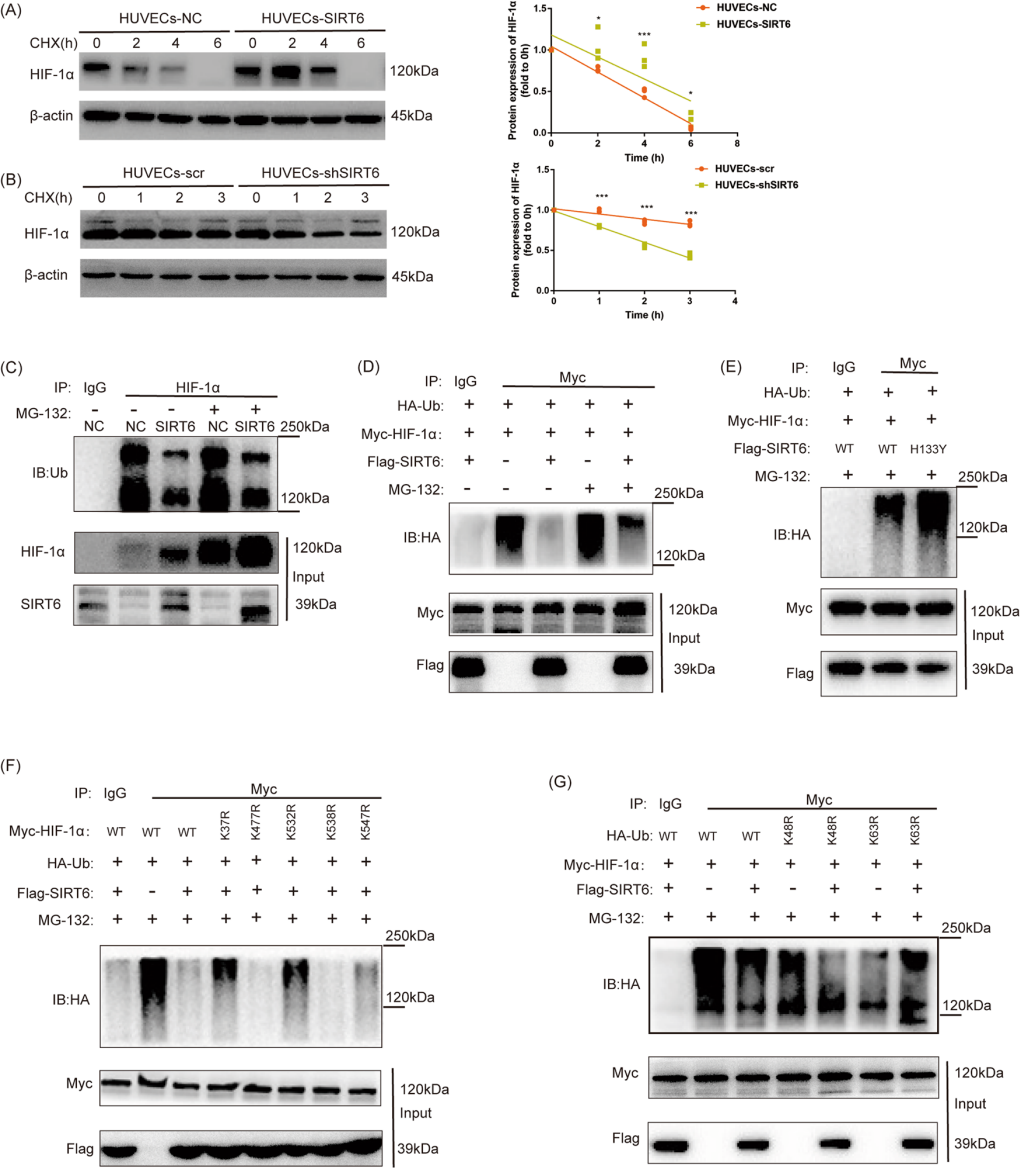
Overexpression of SIRT6 inhibited ubiquitin-proteasome degradation of HIF-1α. (**C**, **D**, **E**, **F**, **G**: Detection of ubiquitination of HIF-1α performed by Co-IP under hypoxia). **A** Overexpression of SIRT6 prevented the degradation of HIF-1α under hypoxia after the treatment of CHX (20 µg/ml for 0–6 h). **B** Downregulation of SIRT6 promoted the degradation of HIF-1α under hypoxia after the treatment of CHX (20 µg/ml for 0–3 h). **C** Ubiquitination of HIF-1α in HUVECs-NC and HUVECs-SIRT6 with or without proteasome inhibitor MG-132 treatment (10 μm, 6 h). **D** SIRT6 promoted deubiquitination of HIF-1α. HUVECs were transfected with HA-Ub, Myc-HIF-1α, and SIRT6-Flag, respectively. **E** Inhibition of histone deacetylase activity of SIRT6 inhibited its deubiquitination effect at HIF-1α. HUVECs were transfected with HA-Ub, Myc-HIF-1α, and SIRT6-Flag (wild type: WT or histone deacetylase activity inhibited mutant: H133Y), respectively. **F** K37 and K532 are the main SIRT6 deubiquitination sites of HIF-1α. HUVECs was transfected with HA-Ub, SIRT6-Flag, and different Myc-HIF-1α mutant (K37R/K477R/K532R/K538R/K547R). **G** SIRT6 promoted the deubiquitination of HIF-1α via Ubiquitin-K63. HUVECs were transfected with SIRT6-Flag, Myc-HIF-1α, and HA-Ub (WT, K48R, and K63R mutant) (*P < 0.05, ***P < 0.001).

Ubiquitin-proteasome system (UPS) is the main mechanism for the stability and degradation of HIF-1α. We investigated the stability of HIF-1α under hypoxia via treating with protein synthesis cycloheximide (CHX) at 20 µg/ml for 0–6 h (after overexpression of SIRT6) or 0–3 h (after downregulation of SIRT6). Then, protein expression of HIF-1α detected at each time point was normalized by its expression at 0 h to draw the degradation curve. The normalized protein expression of HIF-1α showed significant increase after overexpression of SIRT6 at each time point, which means overexpression of SIRT6 increased the stability of HIF-1α (Fig. 4A). The opposite result was also acquired in SIRT6 downregulated HUVECs (Fig. 4B). Furthermore, Co-IP assay was performed to determine the ubiquitination of HIF-1α with or without treatment of proteasome inhibitor MG-132 (10 μm, 6 h). Interestingly, the endogenous ubiquitination of HIF-1α showed a significant decrease after overexpression of SIRT6 both with and without treatment of MG-132 (Fig. 4C). Meanwhile, tagged plasmids (HA-Ub, Myc-HIF-1α, and Flag-SIRT6) was transfected to HUVECs for further verification, and a similar result was acquired (Fig. 4D). We also investigated whether histone deacetylase activity of SIRT6 affects its deubiquitination of HIF-1α by mutant SIRT6 (H133Y). The mutant SIRT6 (H133Y) showed significantly increased ubiquitination of HIF-1α compared with wide type, which indicates histone deacetylase activity of SIRT6 is crucial for the deubiquitination of HIF-1α (Fig. 4E). Subsequently, we investigated possible ubiquitination sites of HIF-1α in HUVECs via LC-MS and four ubiquitination sites were finally confirmed (K37, K477, K532, and K547). Subsequently, we constructed mutant plasmids of these ubiquitination sites of HIF-1α. The ubiquitination was rescued in K37R and K532R mutant, which indicated K37 and K532 were main SIRT6 deubiquitination sites of HIF-1α (Fig. 4F). K48-linkage-specific ubiquitination promotes proteasome-mediated proteolysis, while K63-linkage-specific ubiquitination regulates numerous functions of target proteins such as protein–protein interactions. To research which site of Ubiquitin participated in the deubiquitination effect of SIRT6, we further constructed Ub-K48R and Ub-K63R mutant plasmids. We found a significant repression of the deubiquitination effect of SIRT6 in Ub-K63R but not in Ub-K48R transfected HUVECs (Fig. 4G).

### SIRT6 promoted migration, proliferation, and tube formation ability of HUVECs via regulation of HIF-1α

**Fig. 5:**
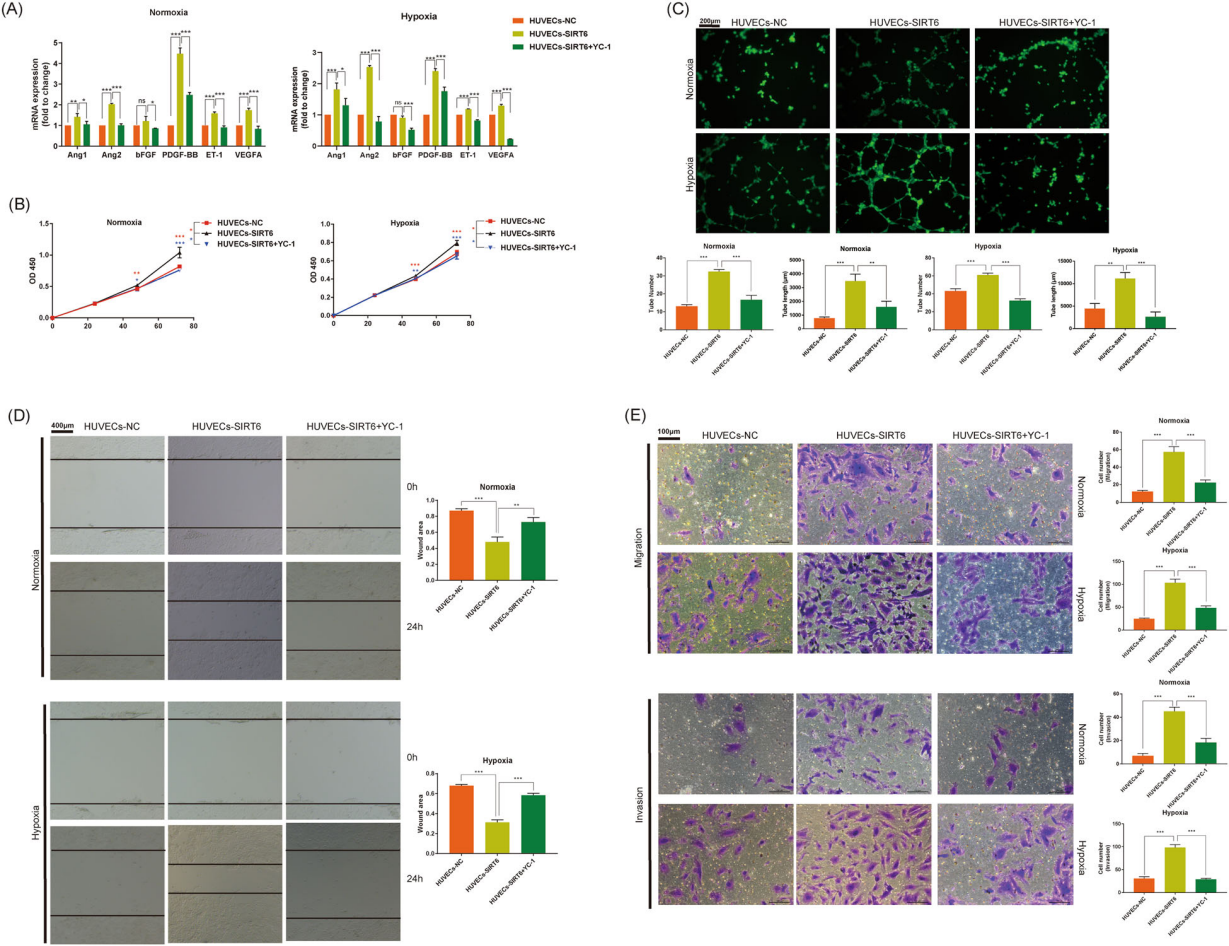
SIRT6 promoted migration, proliferation, and tube formation ability of HUVECs via regulation of HIF-1α. **A** The expression of HIF-1α regulated angiogenic genes. **B** Cell proliferation of HUVECs-NC, HUVECs-SIRT6, and HUVECs-SIRT6 treated with HIF-1α inhibitor YC-1 (1 μm for 0–72 h) under both hypoxia and normoxia, performed by CCK8 assays. **C** Tube formation ability of HUVECs-NC, HUVECs-SIRT6, and HUVECs-SIRT6 pre-treated with HIF-1α inhibitor YC-1 (1 μm for 24 h) under both hypoxia and normoxia (magnification: ×200). **D** Invasion and migration ability of HUVECs-NC, HUVECs-SIRT6, and HUVECs-SIRT6 treated with HIF-1α inhibitor YC-1 (1 μm for 24 h) under both hypoxia and normoxia, performed by transwell assays (magnification: ×400). **E** Migration ability of HUVECs-NC, HUVECs-SIRT6, and HUVECs-SIRT6 treated with HIF-1α inhibitor YC-1 (1 μm for 24 h) under both hypoxia and normoxia, performed by wound healing assay (magnification: ×100) (ns: no significance, *P < 0.05, **P < 0.01, ***P < 0.001).

HIF-1α regulates a series of angiogenic molecules, including angiopoietin (Ang), platelet-derived growth factor (PDGF), vascular endothelial growth factor (VEGF), endothelin (ET), and so on. As expected, overexpression of SIRT6 significantly promoted the expression of Ang1, Ang2, PDGF-BB, ET-1, and VEGFA under both normoxia and hypoxia. In addition, the treatment of HIF-1α inhibitor YC-1 rescued all the promotion (Fig. 5A). Similarly, Overexpression of SIRT6 significantly promoted the proliferation of HUVECs from 48 h both under hypoxia and normoxia. Meanwhile, HIF-1α inhibitor YC-1 successfully rescued the promotion (Fig. 5B). Overexpression of SIRT6 also increased both tube number and length of HUVECs, and YC-1 rescued the increase both under hypoxia and normoxia (Fig. 5C). To investigate the effect of SIRT6 at migration and invasion of HUVECs, transwell assays were performed. We demonstrated overexpression of SIRT6 promoted migration and invasion of HUVECs both under hypoxia and normoxia, and HIF-1α inhibitor YC-1 successfully rescued the promotion (Fig. 5D). The migration ability examined by the wound healing assay showed similar results (Fig. 5E).

### SIRT6 promoted reactive oxygen species via H3K56ac of catalase in HUVECs under oxidative stress

**Fig. 6:**
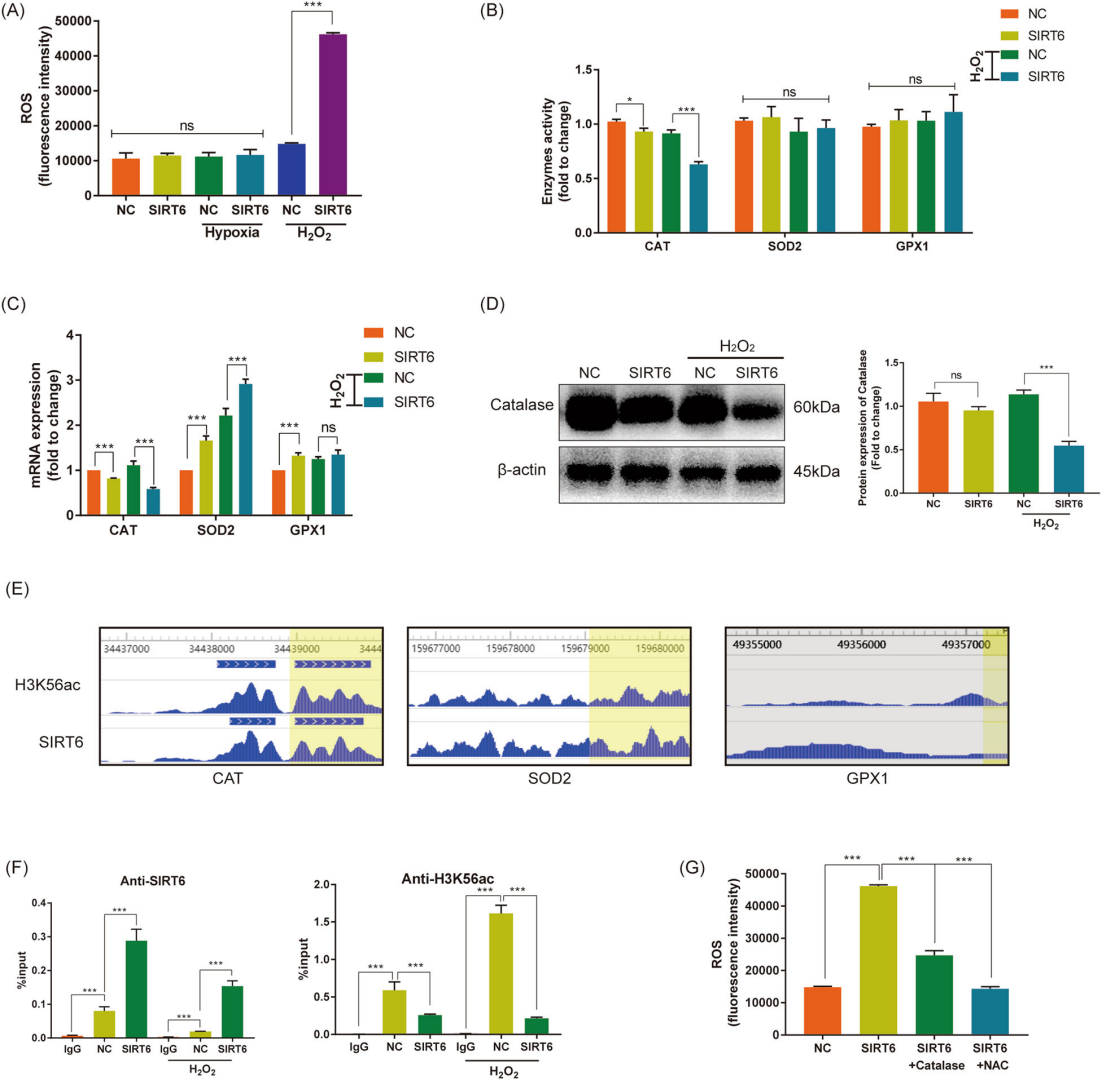
SIRT6 promoted reactive oxygen species via H3K56ac of Catalase in HUVECs under oxidative stress. **A** ROS of HUVECs-NC and HUVECs-SIRT6 under normoxia (normal group), hypoxia, and oxidative stress (treated with 1 mM H_2_O_2_ for 3 h). **B** The enzyme activity of three main ROS scavengers: Catalase (CAT), superoxide dismutase (SOD2), and glutathione peroxidase (GPX1), with or without H_2_O_2_ treatment. **C** The mRNA expression of CAT, SOD2, and GPX1, with or without H_2_O_2_ treatment. **D** Protein expression of Catalase with or without H_2_O_2_ treatment performed by western blotting. **E** SIRT6 and H3K56ac enrichment at the promoter of *CAT*, *SOD2*, and *GPX1* detected in CHIP-sequence datasets GSE102813. **F** CHIP analysis of HUVECs-NC and HUVECs-SIRT6 using IgG, anti-SIRT6, and anti-H3K56ac, respectively. **G** Transfection of pcDNA3.1-Catalase and treatment of N-acetyl-L-cysteine (NAC, 5 mM for 3 h) rescued increased ROS in HUVECs-SIRT6 (ns: no significance, **P < 0.01, ***P < 0.001).

Our previous research had demonstrated SIRT6 promoted ROS in papillary thyroid cancer^15^. ROS is the main product of oxidative stress. To determine the effect of SIRT6 at ROS in HUVECs, DCFH-DA probe was treated and fluorescence intensity was read. The ROS of HUVECs-SIRT6 showed no change compared with HUVECs-NC both normoxia (normal group) and hypoxia. However, in oxidative stress induced by hydrogen peroxide, ROS of HUVECs-SIRT6 was significantly increased compared with HUVECs-NC (Fig. 6A). Catalase, superoxide dismutase and glutathione peroxidase are three main ROS scavengers, their enzyme activities were examined. We found the overexpression of SIRT6 slightly repressed the activity of Catalase, and oxidative stress treatment significantly augmented the repression. No changes in the activity of superoxide dismutase and glutathione peroxidase were determined (Fig. 6B). The mRNA expression of their coding genes (*CAT, SOD2, GPX1*) was detected by RT-qPCR. We found overexpression of SIRT6 significantly inhibited mRNA expression of *CAT*; meanwhile, oxidative stress treatment promoted the inhibition. The mRNA expression of *SOD2* and *GPX1* showed a totally opposite trend in both normal group and oxidative stress group (Fig. 6C). Subsequently, we determined the expression and activity of catalase via western blotting and assay kits, respectively. Similar to the results of RT-qPCR, overexpression of SIRT6 slightly inhibited the protein expression of Catalase, and oxidative stress further promoted the inhibition (Fig. 6D). Considering SIRT6 is a member of the Class III histone deacetylases (HDAC) that have been demonstrated inhibited H3K56ac We consulted the CHIP-sequence datasets GSE102813 which studied SIRT6 depletion in BRAF^V600E^ melanoma cells^16^. We found SIRT6 binds to the promoter of *CAT*, accompanied by a significant H3K56ac peak (Fig. 6E). To research specific mechanism at the transcriptional level, CHIP analysis was performed. The promoter region of *CAT* showed significant enrichment in anti-SIRT6 group compared with anti-IgG group, and the overexpression of SIRT6 promoted enrichment. Oxidative stress further augmented the promotion. Oppositely, overexpression of SIRT6 depleted H3K56ac at the promoter of *CAT*, and oxidative stress further promoted the depletion (Fig. 6F). Finally, we transfected pcDNA3.1-Catalase into HUVECs-SIRT6 and successfully rescued the increase of ROS. In addition, the treatment of ROS scavenger N-acetyl-L-cysteine (NAC, 5 mM for 3 h) also significantly inhibited ROS (Fig. 6G). All these indicated SIRT6 bound to the promoter of *CAT* and depleted H3K56ac, thus oppressed its expression at the transcriptional level. Furthermore, Oxidative stress significantly amplified the effect, and eventually promoted the accumulation of ROS.

### SIRT6 deepened mitochondrial damage of HUVECs via ROS under oxidative stress

Mitochondria is not only the main producing organelle of ROS, but also the damaging target of ROS^17^. The main function of mitochondria is to produce ATP through oxidative respiration. Therefore, we further focused on the effect of SIRT6 on ATP content and oxidative respiration. Overexpression of SIRT6 inhibited ATP content only under oxidative stress, but not under normal group; meanwhile, NAC rescued the inhibition (Figure S2A). Furthermore, we detected the oxygen consumption rate (OCR) of cells via Seahorse XF96 analysis, which is the main index of mitochondria oxidative respiration. Similar to the results of ATP content, SIRT6 significantly inhibited OCR only under the oxidative stress group; meanwhile, NAC rescued the inhibition (Figure S2B).

### SIRT6 promoted apoptosis and repressed cell activity of HUVECs via ROS under oxidative stress

Excessive ROS leads to oxidative stress injury and finally promotes apoptosis of cells. The mitochondrial membrane potential of cells, a landmark in the early stage of apoptosis, was detected by the JC-1 probe. We found overexpression of SIRT6 significantly inhibited mitochondrial membrane potential in the oxidative stress group, but not the normal group; meanwhile, NAC rescued the inhibition (Figure S3A, B). Subsequently, we detected cell apoptosis rate via flowcytometry. Oppositely to the trend of mitochondrial membrane potential, overexpression of SIRT6 significantly promoted the apoptosis rate of HUVECs in oxidative stress group, but not under normal group; meanwhile, NAC rescued the promotion (Figure S3C, D, E). Finally, we examined the cell activity of HUVECs under oxidative stress via CCK8 assay. 1 mM H_2_O_2_ successfully inhibited activity of HUVECs-NC, and overexpression of SIRT6 (HUVECs-SIRT6) further repressed cell activity. NAC also successfully rescued the repression. We further aggravated the oxidative stress by increasing the concentration of H_2_O_2_ to 5 mM, and a similar result was acquired (Figure S3F).

### SIRT6 promoted angiogenesis as well as vessel permeability in vivo

**Fig. 7:**
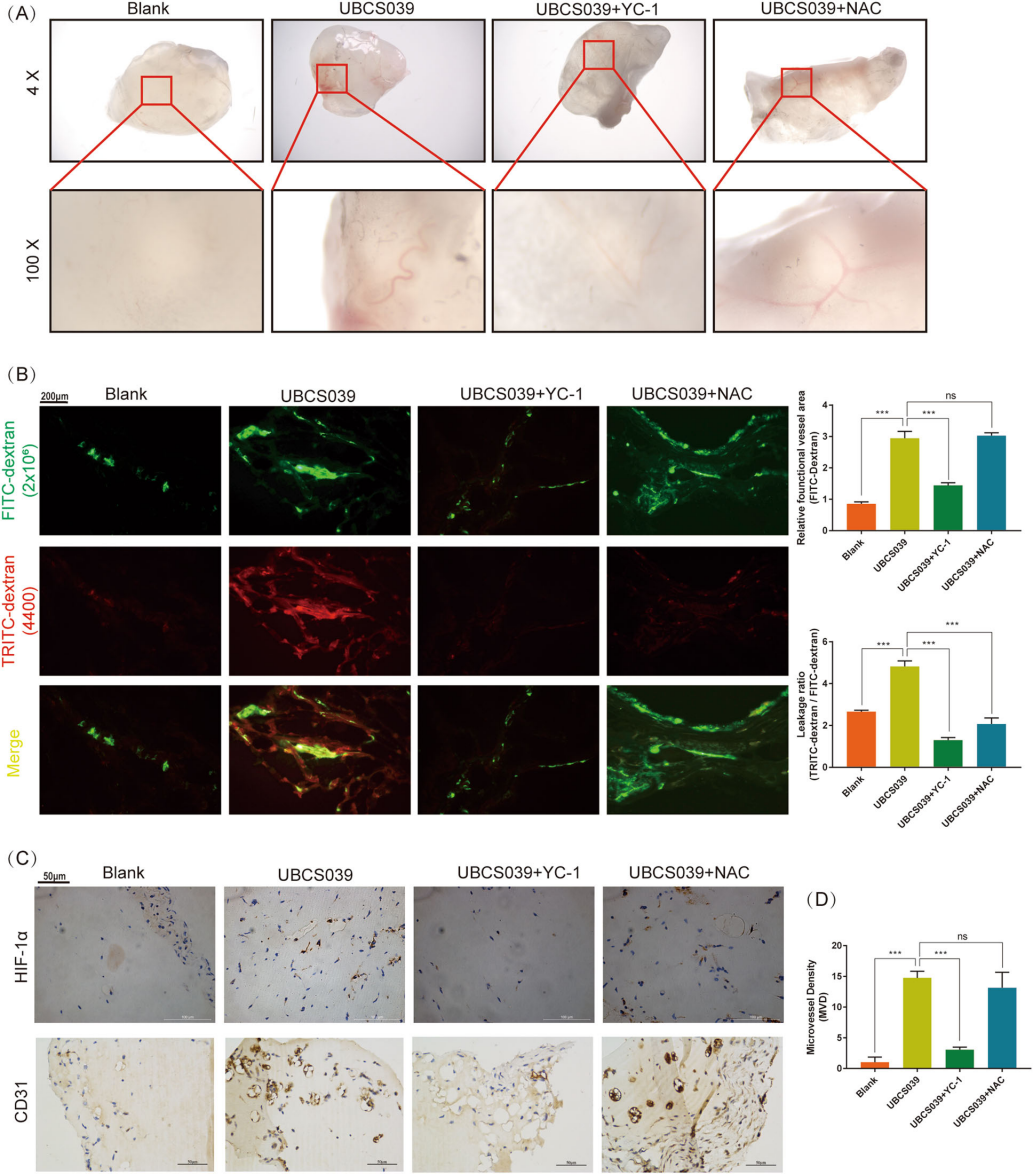
SIRT6 promoted angiogenesis as well as vessel permeability in vivo (*N* = 5, each group). **A** Matrigel plugs deprived from mice after 14 days of subcutaneous implantation. UBCS039: SIRT6 activator, 100 μm; YC-1: HIF-1α inhibitor, 2 μm; NAC: ROS scavenger, 10 μm. All drugs were blended with Matrigel gel before implantation. **B** FITC-dextran (2 × 10^6^ MW) accompanied by TRITC-dextran (4400 MW) was injected before the scarification of mice. FITC-dextran (2 × 10^6^ MW): functional vessel, TRITC-dextran (4400 MW): leaky blood cells from vessels. TRITC-dextran/FITC-dextran area (leakage ratio): vessel permeability (magnification: ×100). **C** Expression of HIF-1α and microvascular density (MVD, marked by CD31) of all matrigel plugs performed by IHC (magnification: ×400) (ns: no significance, ***P < 0.001).

As researched above, SIRT6 simultaneously promoted the expression of HIF-1α as well as ROS of HUVECs. On one hand, HIF-1α is an important transcription factor of angiogenesis and induced proteins include VEGF, PDGF, Ang, and so on^18,19^. On the other hand, ROS promoted oxidative stress injury of HUVECs. SIRT6 seemed to have a complex effect at the growth of vessels. To investigated the role of SIRT6 in angiogenesis, Matrigel plug assay was performed. SIRT6 activator UBCS039 (100 μm, blended in matrigel plugs) significantly promoted angiogenesis in Matrigel plugs compared with blank group. HIF-1α inhibitor YC-1 (2 μm, blended in matrigel plugs) but not ROS scavenger NAC (10 μm, blended in matrigel plugs) significantly rescued the promotion of SIRT6 activator (Fig. 7A). Subsequently, FITC-dextran (2 × 10^6^ MW) accompanied with TRITC-dextran (4400 MW) was used to present functional vessel as well as leaky blood cells from vessels. The ratio of TRITC-dextran/FITC-dextran area (leakage ratio) was applied for assessing vessel permeability. We demonstrated SIRT6 activator promoted vessel permeability, whereas both HIF-1α inhibitor and ROS scavenger significantly rescued the promotion (Fig. 7B). Finally, IHC was performed to examine the expression of HIF-1α and MVD. As expected, SIRT6 activator promoted expression of HIF-1α and MVD in matrigel plugs, HIF-1α inhibitor but not ROS scavenger rescued the promotion (Fig. 7C). These results indicated SIRT6 promoted angiogenesis and vessel permeability via HIF-1α. In addition, SIRT6-induced ROS only promoted vessel permeability, but not angiogenesis.

## Discussion

Previous researches had demonstrated that knockdown of SIRT6 promoted the development of AS, and inferred SIRT6 works as a protective factor. Our research seems to contradict previous studies. However, we suggested the contradiction came from the focus of different developing stages of AS. All our research focused on the pathology of formed plaque, but not the formation of plaque. We determined SIRT6 was overexpressed in unstable carotid plaque compared with stable carotid plaque. The overexpression of SIRT6 in mice model also promoted angiogenesis and IPH in plaque via promoting HIF-1α.

HIF-1α is an important transcription factor of angiogenesis that stably accumulates in hypoxia, whereas degrades rapidly after synthesis in normoxia. In AS, the local O_2_ diffusion from the arterial lumen could be insufficient because of intimal thickening and inflammation, thus created hypoxia environment for overexpression of HIF-1α^20^. HIF-1α activates multiple genes contributing to the angiogenic process^21^. HIF-1α induced proteins include vascular endothelial growth factor (VEGF) and basic fibroblast growth factor (bFGF), which promote vascular permeability and endothelial cell growth, respectively^22^. Other secreted factors, such as platelet-derived growth factor (PDGF), angiopoietin-1 (ANG-1), and angiopoietin-2 (ANG-2), facilitate chemotaxis, while ephrin signaling guides newly formed blood vessels by controlling motility and cell–cell adhesion^18,19^. Previous researches have demonstrated HIF-1α promoted the development of AS via regulating differentiation of macrophages, destabilization of plaque, growth of vasorum^23–25^.

To research the interaction of SIRT6 and HIF-1α, SIRT6 overexpressed HUVECs was generated. We found SIRT6 promoted the protein expression of HIF-1α both under hypoxia and normoxia. Similar result was also acquired in VSMC and papillary thyroid cancer cell lines TPC-1 and B-CPAP^26^, which indicated a universal promotion of SIRT6 at HIF-1α. We further investigated the mRNA expression of HIF-1α and no change was found. We inferred SIRT6 promoted expression of HIF-1α at the protein level. As ubiquitin-proteasome system (UPS) is the main way for the degradation of HIF-1α; meanwhile, SIRT6 protein was reported to interact with HIF-1α protein^27^. Therefore, we inferred SIRT6 regulated the UPS of HIF-1α. As expected, overexpression of SIRT6 promoted stability whereas inhibited ubiquitination of HIF-1α in HUVECs. Through LC/MS and mutant vectors, K37 and K532 were finally confirmed as the main SIRT6 deubiquitination sites at HIF-1α. Ub-K63 was confirmed as the main Ubiquitin site. We also demonstrated histone deacetylase activity of SIRT6 is crucial for the deubiquitination of HIF-1α. These results indicated SIRT6 promoted the expression and stability of HIF-1α via deubiquitination. Furthermore, we investigated the effect of SIRT6 on biological function of HUVECs. As expect, SIRT6 promoted the expression of various angiogenesis factors via HIF-1α, including Ang1, Ang2, PDGF-BB, VEGFA, and ET-1. SIRT6 promoted invasion, migration, proliferation, and tube formation ability of HUVECs under both normoxia and hypoxia; meanwhile, inhibition of HIF-1α rescued all promotion. These results demonstrated SIRT6 played its angiogenic role via regulating HIF-1α.

In our previous research, we also found SIRT6 increased ROS in papillary thyroid cancer B-CPAP^15^. In addition, other researches on the effect of SIRT6 on ROS were contrasting in different cells. Overexpression of SIRT6 attenuated hypoxia-enhanced glycolysis and ROS generation in human osteoblastic cells^28^. Whereas in glioma cells, SIRT6 was reported to increase ROS, thus reduced cell survival under H_2_O_2_ treatment^29^. We examined ROS in HUVECs-SIRT6, whereas no increase was found under both normoxia and hypoxia. Considering oxidative stress model is the main approach to learn ROS as well as main pathogenesis of AS, we re-examined the ROS in H_2_O_2_-induced oxidative stress^30,31^. Encouragingly, SIRT6 significantly promoted ROS of HUVECs in the oxidative stress model. Catalase, superoxide dismutase, and glutathione peroxidase are three main ROS scavengers, thus we examined the expression and activity^17^. Furthermore, we demonstrated SIRT6 bound to the promoter of Catalase and depleted H3K56ac, thus inhibited its expression and activity in the oxidative stress model. Meanwhile, a similar but obviously weakened effect was detected in normal group, which explained the unchanged ROS.

Mitochondria is the main place producing the ROS and the most sensitive position reflected to the effect of ROS^17^. Mitochondria is the main organelle of aerobic respiration and ATP production; thus, we examined these two indexes in HUVECs. Overexpression of SIRT6 inhibited both OCR and ATP content under oxidative stress, and NAC rescued the inhibition. These results indicated SIRT6 promoted mitochondrial dysfunction via promoting ROS. As the high concentration of ROS is also the signal which starts the apoptosis^32,33^. We also demonstrated SIRT6 promoted apoptosis, as well as early event of apoptosis-decrease of mitochondrial membrane potential via promoting ROS. Finally, we learned the cell viability under oxidative stress. Overexpression of SIRT6 significantly inhibited cell viability, whereas ROS scavenger NAC rescued the inhibition. Which indicated SIRT6 represses cell viability under oxidative stress via promoting ROS.

On one hand, SIRT6 promoted angiogenesis under normoxia and hypoxia; on the other hand, SIRT6 also induced apoptosis and mitochondrial dysfunction under oxidative stress. These results seem contradictory but can be well explained combined with the pathogenesis of AS. The local O_2_ diffusion from the arterial lumen could be insufficient because of intimal thickening and inflammation, thus created a hypoxia environment for overexpression of HIF-1α. Accompanied by the deubiquitination effect of SIRT6, HIF-1α was rapidly accumulated. The overexpression of HIF-1α further promoted various activates angiogenic genes, thus promoted the angiogenesis in plaque. Meanwhile, SIRT6 induced the accumulation of ROS via repressing Catalase. ROS-induced vascular endothelial injury led directly to endothelial barrier dysfunction^34,35^. We suggested ROS damaged HIF-1α-promoted vessels in plaque, thus led to IPH. Our suggestion was justified by the Matrigel plug assay. SIRT6 promoted HIF-1α is responsible for neovascularization as well as vascular permeability. SIRT6 promoted ROS showed no effect in neovascularization but contributed to vascular permeability.

In conclusion, our research demonstrated SIRT6 promoted angiogenesis in carotid artery plaque via promoting HIF-1α. Subsequently, the overexpression of HIF-1α further promoted various activates angiogenic genes, thus promoted angiogenesis in plaque. In addition, SIRT6 also promoted ROS under oxidative stress, which a common environment of AS^30^. The ROS further promoted endothelial injury of carotid artery as well as damage of new vessels in plaque. All effects of SIRT6 finally promoted the instability of carotid plaque.

## Supplementary information

Supplementary Figure Legends

Figure S1

Figure S2

Figure S3

Table S1

## Data Availability

The datasets used and analyzed during the current study are available from the corresponding author on reasonable request.
