# Supplementary material for: SIRT6 promotes angiogenesis and hemorrhage of carotid plaque via regulating HIF-1α and reactive oxygen species
Source: Cell Death Dis. 2021 Jan 12;12(1):77. doi: 10.1038/s41419-020-03372-2 (PMC7804142; doi:10.1038/s41419-020-03372-2)
Supplement: Supplementary file 1 — Supplementary Figure Legends [file 41419_2020_3372_MOESM1_ESM.docx]

Figure S1. Co-location of SIRT6 and CD31.

Figure S2. SIRT6 deepened mitochondrial damage of HUVECs via ROS under oxidative stress. A. ATP content of HYVECs-NC, HUVECs-SIRT6, and HUVECs-SIRT6 treated with NAC (5mM for 3h), with or without H_2_O_2_ treatment (1mM, 3h). B. Oxygen consumption rate (OCR) of HYVECs-NC, and HUVECs-SIRT6. and HUVECs-SIRT6 treated with NAC (with or without treatment of 1mM H_2_O_2_ for 3h).

Figure S3. SIRT6 promoted apoptosis and repressed cell activity of HUVECs via ROS under oxidative stress. A. The mitochondrial membrane potential of HUVECs cells (NC, SIRT6, SIRT6 treated with 5mM NAC for 3h) with or without H2O2 treatment (1mM, 3h), (Captured by fluorescence microscope, magnification 400x). B. The mitochondrial membrane potential of HUVECs cells described above, quantitatively detected by a fluorescent microplate reader (aggregate: 585/590mm, monomer:514/529mm, showed as aggregate/monomer). C. Cell apoptosis of HUVECs-NC and HUVECs-SIRT6 performed by flow cytometry. D. Cell apoptosis of HUVECs-NC, HUVECs-SIRT6, and HUVECs-SIRT6 treated with NAC (all treated with 1mM H2O2 for 3h). E. Quantitative analysis of apoptosis rate in panel C and D. F. Cell activity of HUVECs-NC, HUVECs-SIRT6, HUVECs-SIRT6 treated with NAC performed by CCK8 assay (with or without treatment of 1/5mM H2O2 for 3h). (ns: no significance, **P＜0.01, ***P＜0.001).
