# Supplementary figures and images for: SIRT6 promotes angiogenesis and hemorrhage of carotid plaque via regulating HIF-1α and reactive oxygen species
Source: Cell Death Dis. 2021 Jan 12;12(1):77. doi: 10.1038/s41419-020-03372-2 (PMC7804142; doi:10.1038/s41419-020-03372-2)

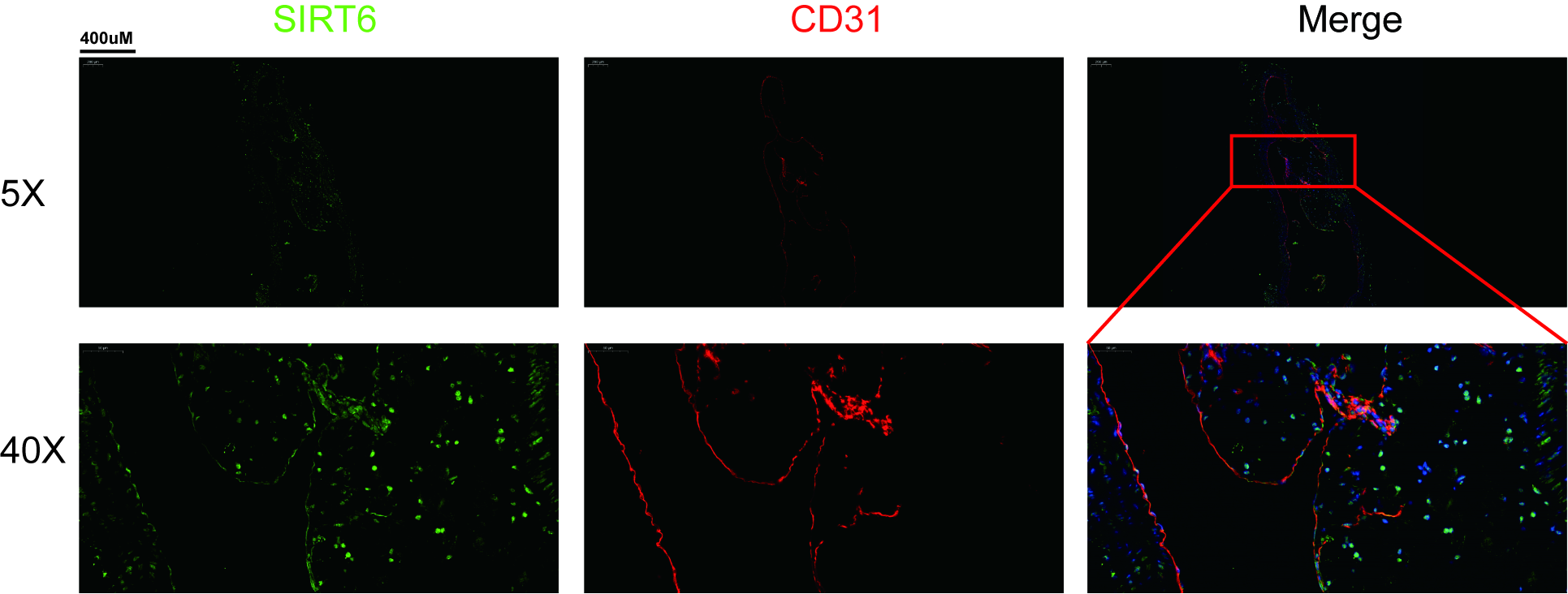

Supplement: Supplementary file 2 — Figure S1 [file 41419_2020_3372_MOESM2_ESM.tif]

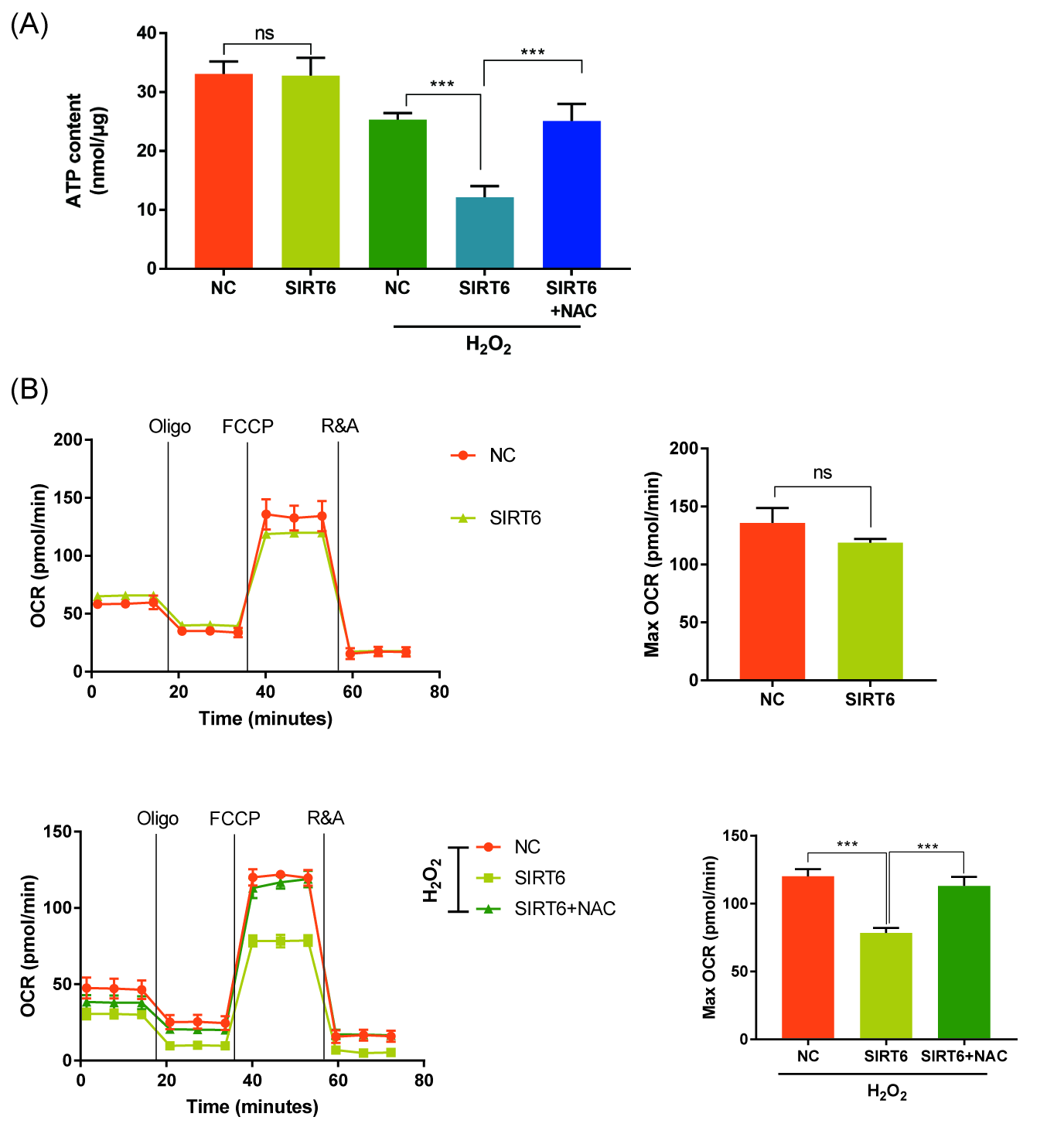

Supplement: Supplementary file 3 — Figure S2 [file 41419_2020_3372_MOESM3_ESM.tif]

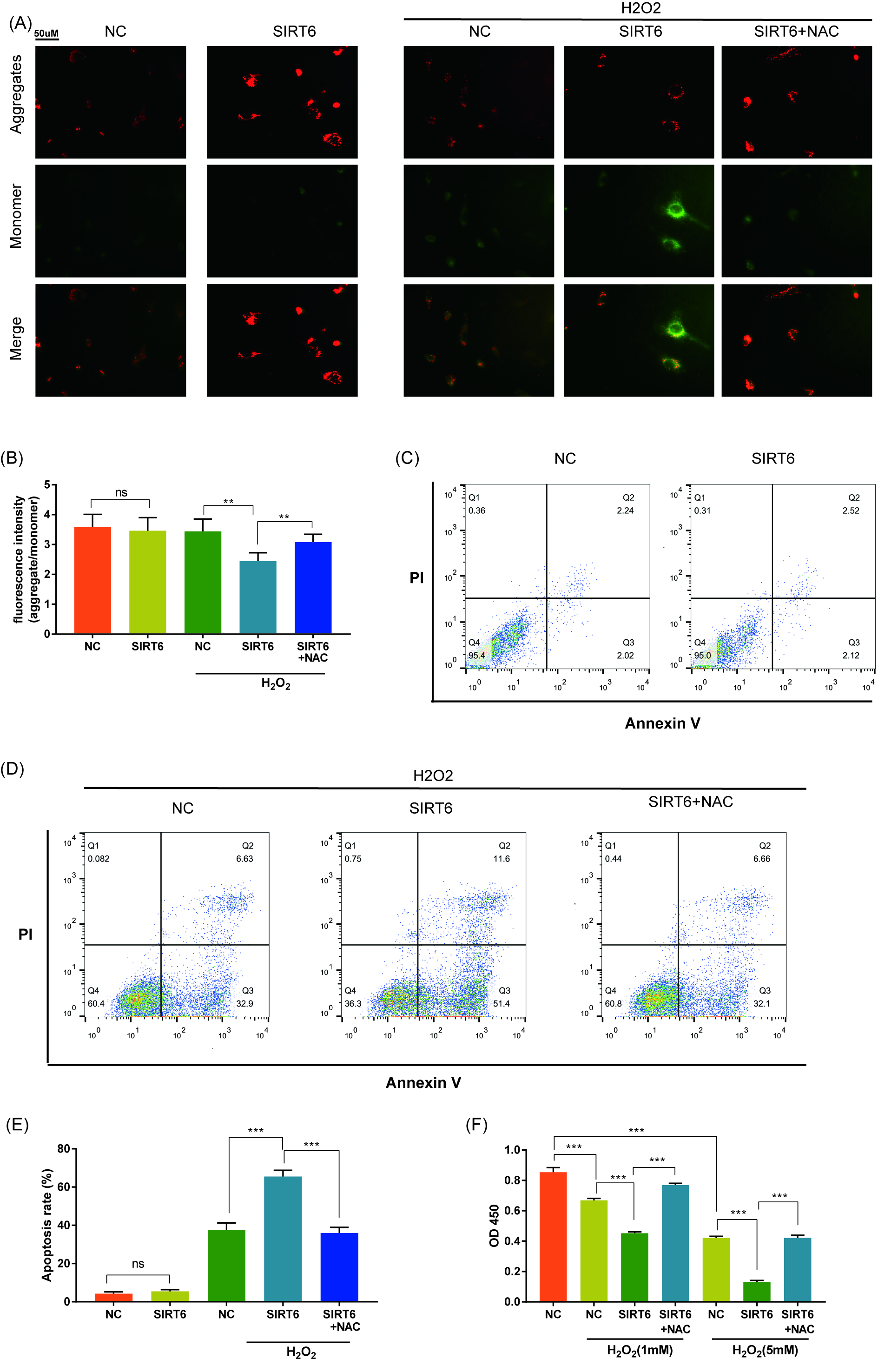

Supplement: Supplementary file 4 — Figure S3 [file 41419_2020_3372_MOESM4_ESM.tif]
