# Supplementary material for: SIRT6 promotes angiogenesis and hemorrhage of carotid plaque via regulating HIF-1α and reactive oxygen species
Source: Cell Death Dis. 2021 Jan 12;12(1):77. doi: 10.1038/s41419-020-03372-2 (PMC7804142; doi:10.1038/s41419-020-03372-2)
Supplement: Supplementary file 5 — Table S1 [file 41419_2020_3372_MOESM5_ESM.docx]

Primers for RT-qPCR

| **Gene** | **Forward Primer** | **Reverse Primer** |
| --- | --- | --- |
| **SIRT6** | GCACCGTGGCTAAGGCAAGG | GTGATGGACAGGTCGGCGTTC |
| **HIF1A** | GAACGTCGAAAAGAAAAGTCTCG | CCTTATCAAGATGCGAACTCACA |
| **Ang1** | AGCGCCGAAGTCCAGAAAAC | TACTCTCACGACAGTTGCCAT |
| **Ang2** | AACTTTCGGAAGAGCATGGAC | CGAGTCATCGTATTCGAGCGG |
| **bFGF** | AGAAGAGCGACCCTCACATCA | CGGTTAGCACACACTCCTTTG |
| **PDGF-BB** | CTCGATCCGCTCCTTTGATGA | CGTTGGTGCGGTCTATGAG |
| **ET-1** | AGAGTGTGTCTACTTCTGCCA | CTTCCAAGTCCATACGGAACAA |
| **VEGFA** | AGGGCAGAATCATCACGAAGT | AGGGTCTCGATTGGATGGCA |
| **CAT** | TGGAGCTGGTAACCCAGTAGG | CCTTTGCCTTGGAGTATTTGGTA |
| **GPX1** | CAGTCGGTGTATGCCTTCTCG | GAGGGACGCCACATTCTCG |
| **SOD2** | GCTCCGGTTTTGGGGTATCTG | GCGTTGATGTGAGGTTCCAG |
| **Actin** | AGCGAGCATCCCCCAAAGTT | GGGCACGAAGGCTCATCATT |
